# Effect of Plasma-Derived Exosomes of Refractory/Relapsed or Responsive Patients with Diffuse Large B-Cell Lymphoma on Natural Killer Cells Functions

**DOI:** 10.22074/cellj.2020.6550

**Published:** 2019-09-08

**Authors:** Nasrin Zare, Shaghayegh Haghjooy Javanmard, Valiollah Mehrzad, Nahid Eskandari, Alireza Andalib

**Affiliations:** 1 Department of Immunology, School of Medicine, Isfahan University of Medical Sciences, Isfahan, Iran; 2 Department of Physiology, School of Medicine and Applied Physiology Research Center, Cardiovascular Research Institute, Isfahan University of Medical Sciences, Isfahan, Iran; 3 Department of Hematology and Medical Oncology, Isfahan University of Medical Sciences, Isfahan, Iran

**Keywords:** Cytotoxicity, Diffuse Large B-Cell Lymphoma, *hsa-miR-155-5p*, Interferon-Gamma, Proliferation

## Abstract

**Objective:**

The purpose of this study was to investigate effect of plasma-derived exosomes of refractory/relapsed or responsive diffuse large B-cell lymphoma (DLBCL) patients on natural killer (NK) cell functions.

**Materials and Methods:**

In this cross-sectional and experimental study, NK cells were purified from responsive patients (n=10) or refractory/relapsed patients (n=12) and healthy donors (n=12). NK cells were treated with plasma-derived exosomes of responsive or refractory/relapsed patients. We examined the expression levels of *hsa-miR-155-5p, hsa- let-7g-5p, INPP5D (SHIP-1)* and *SOCS-1* in NK cells quantitative reverse transcription-polymerase chain reaction (qRT-PCR). Percentages of NK cells expressing CD69, NKG2D and CD16, NK cell cytotoxicity and NK cell proliferation (using flow-cytometry) as well as interferon-gamma (IFN-γ) level in the supernatant of NK cells using ELISA were also investigated.

**Results:**

We observed an increased level of *hsa-miR-155-5p* and a decreased level of *SOCS-1* in NK cells treated with exosomes compared to untreated NK cell in healthy donors and DLBCL patients. An increase in *hsa-miR-155-5p* level was associated with an increased level of IFN-γ in healthy donors. The decreased levels of hsa-let-7g-5p were observed in NK cells treated with exosomes in comparison with untreated NK cells in DLBCL patients (P<0.05). There was no significant difference in the percentage of CD69^+^NK cells and NKG2D^+^ NK cells in the absence or presence of exosomes of DLBCL patients in each group. Furthermore, we observed significant reduction of NK cell proliferation in DLBCL patients and healthy donors in the presence of exosomes of refractory/relapsed patients (P<0.05). A significant decrease was observed in cytotoxicity of NK cell in patients with DLBCL treated with exosomes of responsive patients.

**Conclusion:**

Our findings demonstrated adverse effect of plasma-derived exosomes of DLBCL patients on some functions of NK cell. It was also determined that low NK cell count might be associated with impaired response to R-CHOP and an increased recurrence risk of cancer.

## Introduction

The most common high-grade form of non-hodgkin lymphoma (NHL) is diffuse large B-cell lymphoma (DLBCL) accounting for more than 30-40% of new cases. B-cells are divided into either indolent (prolonged survival but generally incurable) or aggressive (rapid growth but potentially curable). DLBCL is an aggressive type of lymphoma which can be cured with rituximab, cyclophosphamide, doxorubicin hydrochloride (hydroxydaunomycin), vincristine sulfate (oncovin) and prednisone (R-CHOP). More than half of patients experience complete responses (CRs) and approximately 30% have partial responses (PRs). Despite the advance in treatment, relapsed and refractory disease represent a major treatment challenge; thus, about one- third of patients are either refractory to the treatment or experience relapse (1). Hence, it is necessary to optimize front-line therapy, investigate the physiologic and immunologic circumstances of the patients and develop more effectively salvage strategies (2). 

Natural killer (NK) cells are differentiated from bone marrow and include 5-15% of all peripheral blood mononuclear cells (PBMC). NK cells are defined as large granular lymphocytes expressing CD3-CD19CD56+. NK cells contribute to immune surveillance without prior immunization or major histocompatibility complex (MHC) restriction, as a major component of innate immunity. They induce cytotoxicity or secretion of cytokine/chemokine against infected cells, malignant cells and stressed cells (3). Interferon- gamma (IFN-γ) produced by NK cells is a critical cytokine for the clearance of infectious pathogens and tumor surveillance. Efficient elimination of tumor cell generally requires collaboration between activating and inhibitory receptors. These receptors, including NKp30, NKp46, NKG2D, DNAM-1 and the inducible co-stimulatory molecule CD137 (4-1BB), contribute to antitumor immunity (4). NK cells also express CD16 (Fc.RIIIA), a low-affinity Fc. receptor which can eliminate tumor cells bound to antibodies. CD16 marker is expressed on the cytotoxic CD56dim NK-cell subset, which constitutes about 90% of peripheral NK cells (5). NK cells express activating type of IIIA Fc receptor (FcR.IIIa; CD16a) on their surface. Thus, NK cell-mediated antibody-dependent cellular cytotoxicity (ADCC) occurs through binding to Antibody- coated target cells, leading to NK-cell activation and degranulation (6). 

Previous studies have shown that NK cell activation by IL2, IL-12, IL-15 and IL-18 leads to an increase in the expression levels of *hsa-miR-155-5p* and *hsa-let-7g-5p*. These cytokines activate signal transducer and activator of transcription (STATS). Then, activation of JAK/STATs triggers the suppressor of cytokine signaling (SOCS) proteins, especially SOCS-1, which is a negative regulator of this pathway and it inhibits activation of STATs. Additionally, *hsa-miR-155-5p* directly inhibits SOCS-1 expression. It seems that *hsa-miR155- 5p* could regulate activation of the NK cells by inhibiting *SOCS-1* (7). 

Furthermore, NK cells stimulated by CD16 or IL-12 and IL-18 induces an increase in the *hsa-miR-155-5p* expression. Overexpression of *hsa-miR-155-5p* targets Src homology 2 domain-containing Inositol 5'-phosphatase (SHIP-1) as a negative regulator, consequently up- regulating phosphatidylinositol-3 kinase and enhancing IFN-γ production (8). IFN-γ is a critical cytokine for tumor surveillance. Therefore, understanding molecular pathways of IFN-γ expression could lead to identifying potential therapeutic targets for chronic inflammation and/ or cancer. In this regard, *miR-155* could play an important role in NK cell activation, NK cell cytotoxicity and NK cell immunotherapy (9). 

NKG2D is a member of CD94/NKG2 family of C-type lectin-like receptors. NKG2D is expressed by NK cells and connected to the MHC class I-related chain (MIC) A, MICB, and UL16-binding proteins (ULBPs). These proteins are expressed in the conditions of stress and disease, like cancer. Therefore, NKG2D with its ligands plays a critical role in immunosurveillance of cancer. A reduction of NKG2D ligands results in an impaired susceptibility to NKG2D-mediated cytotoxicity and systemic down-regulation of NKG2D in NK cells of cancer patients (10).

Exosomes are membrane nano-vesicles (30-100 nm) released by most of the cell types in biological fluids such as urine, serum and plasma. Exosomes are involved in both physiological and pathophysiological processes such as coagulation, immune stimulation or suppression, delivery of proteins and genetic material, cell-free viral infection, tumorigenesis and tumor immune escape. Exosomes from different sources contain various types of proteins, lipid classes and nucleic acids. On the other hand, due to the endosomal origin, they have similar protein and lipid combinations. Interestingly, molecular content of exosomes in the sera of cancer patients is different from other exosomes and this profile can induce or suppress immune responses. Exosomes carry genetic information in the form of DNA, mRNA and microRNA; therefore, they can potentially induce genetic changes in target cells (11). 

microRNAs (miRs) including *hsa-miR-155-5p* play significant regulatory roles in proliferation, differentiation, signal transduction, immune responses and carcinogenesis (8). Some evidences showed that elevated expression levels of *hsa-miR-155-5p* in the serum and exosomes isolated from the patients can increase the occurrence of lymphoma, such as DLBCL (12, 13). Furthermore, studies showed an increased expression levels of *hsa-miR-155-5p* and *hsa-let-7g- 5p* in plasma-derived exosomes of patients with chronic lymphoblastic leukemia (CLL) (14). 

*Let-7* family exerts effective anti-tumor and anti- proliferative activities by repressing several oncogenes and key regulators of the cell cycle, cell differentiation and apoptotic pathways. This family is down-regulated in a number of human cancers such as lung, colon, ovarian and breast cancers. Therefore the restoration of *let-7* expression might inhibit cancer growth (15, 16). *Let-7* family contributes to development, muscle formation, cell adhesion and gene regulation in physiological condition. A number of studies have shown that *let-7* family is down-regulated in several types of cancer, including lung cancer, colon cancer and Burkitt’s lymphoma (17). Recent studies have indicated that *hsa-let-7g-5p* can prevent cell invasion and metastasis in gastric and breast cancers. A high *hsa-let-7g-5p* expression might correlate with a lower risk of cancer recurrence in patients with advanced pathological stage (18). 

The purpose of this study was to determine whether exosomes isolated from plasma of patients with DLBCL contribute to NK cell activation or suppression. Therefore, we evaluated their effects on some phenotypical and functional attributes of NK cells from DLBCL patients.

## Materials and Methods

### Subjects

This investigation was a cross-sectional and experimental study. Patients were consecutively selected from the Cancer Referral Centers (Isfahan, Iran). Samples of peripheral blood were obtained from responsive patients with DLBCL (response to R-CHOP, n=10), refractory/relapsed patients with DLBCL (resistant to R-CHOP, n=12) and healthy people (n=12). The mean age of patients was 43.15 ± 11.76 years (mean ± SD); 54.54% of patients were male and 45.45% were female. 

The responsive patients were those who achieved complete remission for 6-12 months after completion of the R-CHOP therapy. The refractory patients were those who failed to respond to six cycles of R-CHOP, as the first-line treatment (n=7). Relapsed patients with DLBCL were those who experienced a relapse at least over a year period after R-CHOP therapy (n=5). The patients who received other chemotherapies, or they had a low- grade DLBCL and/or other different types of NHL, were excluded from the study. 

All subjects signed an informed consent form approved by the Isfahan University of Medical Sciences (Isfahan, Iran). The clinical files and laboratory findings of the patients were reviewed to obtain different characteristics such as age, sex, disease stage, performance statue, nodal/ extra-nodal disease, international prognostic index (IPI) score, serum lactate dehydrogenase (LDH) level, Ki-67 proliferation index (Ki-67 PI) and response to treatment. Immunohistochemically, all patients with DLBCL were non-germinal center B-cell (GCB)-like subtype (CD20^+^CD10^-^BCL-6^-^). The demographic and clinical data of patients with DLBCL were recorded. The samples were carried to the laboratory and used for experiments immediately after processing. 

### Peripheral blood specimen

Blood was drawn into EDTA-containing tubes (10 ml). Peripheral blood mono-nuclear cells (PBMCs) were separated in a Ficoll-Hypaque gradient and they were immediately used for the experiments. Plasma aliquots were either processed for exosomes isolation or stored at -70°C. 

### Preparation of plasma and isolation of exosomes using ExoSpin Exosome purification Kit

Plasma was diluted with an equal volume of sterile phosphate buffered saline (PBS) to decrease viscosity. Since some exosomes might be trapped within the clot when the serum is prepared, EDTA-plasma samples were used rather than serum samples. On the other hand, heparin-plasma samples could facilitate formation of exosome-heparin complexes and aggregation of exosomes, as previously reported (19).

Then plasma was centrifuged at 300 g, 4°C for 10 minutes. It was transferred to the new tube without pellet contamination and centrifuged for 30 minutes at 2000 g, 4°C. The resulting supernatant was centrifuged for a further 30 minutes at 16500 g, 4°C. Plasma was centrifuged by differential centrifugations at increasing speed (300-16500 g) to eliminate large dead cells, large cell debris, platelets, subcellular fragments and larger microvesicles (20).

**Fig.1:**
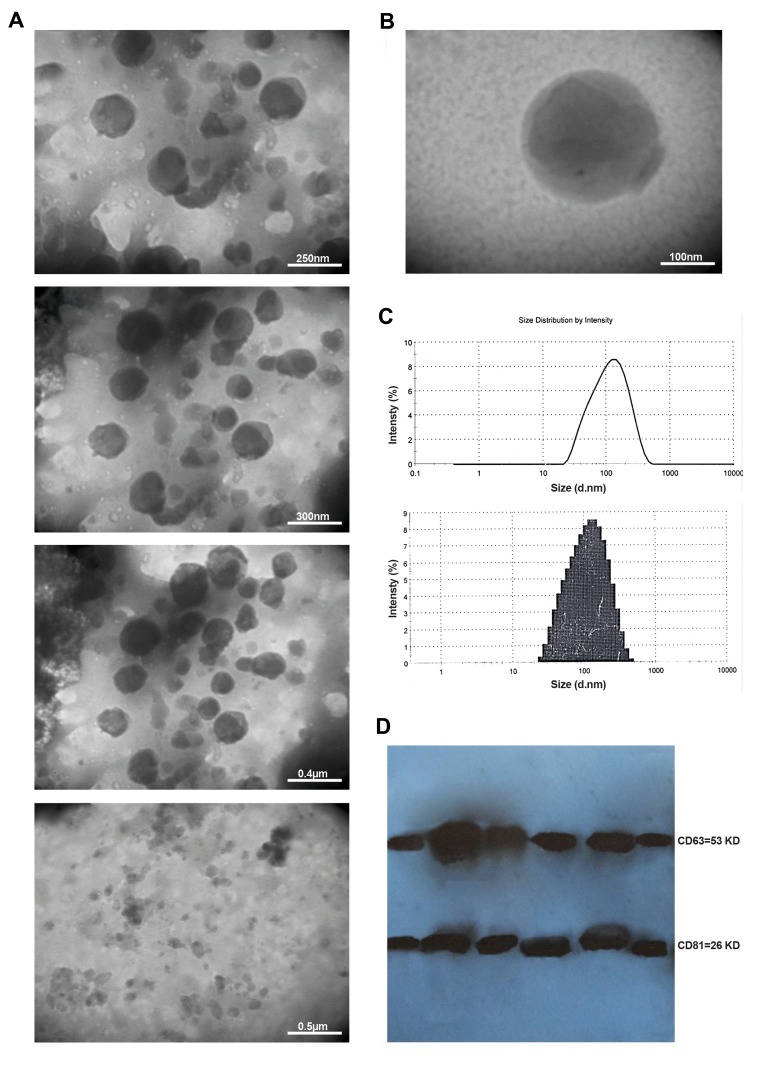
Properties of plasma-derived exosome of DLBCL patients. **A.** Effects of differential centrifugation and ultrafiltration of plasma on isolated exosomes, **B.** The representative TEM image of plasma-derived exosomes (exosomes size: 100 nm), **C.** Size of the all particles in the pellets was determined using a Zetasizer. The z-average particle size was 90.18 nm in diameter, and **D.** The lysed exosomes were separated using polyacrylamide gel electrophoresis and then transferred to the nitrocellulose membrane. The membrane was probed using anti-CD63, anti-CD81 as well as anti-histone H3 and ECL Western blotting systems. DLBCL; Diffuse large B-cell lymphoma and TEM; Transmission electron microscopy.

The supernatant was passed through a 0.22 µm filter and collected in a fresh tube. Ultrafiltration using 0.2 µm filter was performed to remove larger vesicles (above 200 nm) and thrombocytes (about 1-2 µm) remaining in plasma even after differential centrifugation. Apart from thrombocytes and microvesicles, other “contaminating” elements such as lysosomes, mitochondria, nucleic acid- protein aggregates and even bacteria may be present in plasma as seen by by transmission electron microscopy (TEM) (Fig .1A). Ultrafiltration removes the majority of these contaminants. 

Two milliliter aliquots of plasma were processed accordingto the manufacturer’s instructions (Cell Guidance Systems, USA). Briefly, the 1 ml volume of Buffer A was added toeach sample and the sample was vortexed. Samples wereincubated at 4°C for 1 hour and they were next centrifuged for1 hour at 16500 g. Then, the supernatant was discarded andeach pellet was resuspended in 200 µl PBS. The resuspendedpellets were applied to ExoSpin columns and centrifuged at50 g for 60 seconds. The elution was discarded and a further200 µl of PBS was applied to each column. It was centrifugedat 50 g for 60 seconds and the elution containing exosomes was stored at -80°C. 

### Size determination of plasma-derived exosomes

Size of the isolated exosomes was determined using a Zetasizer (Malvern Zen 3600 Instruments, UK) according to the manufacturer’s instructions. The exosomes isolated by the ExoSpin kit were diluted 1:100 in PBS to determine their size. 

### Transmission electron microscopy

Morphology of the exosomes was evaluated by TEM using negative staining. Carbon-coated copper grids were placed on top of 5-10 µl sample drops for 20 minutes and they were fixed by 2% paraformaldehyde. The grids were then washed in the distilled water drops three times for 5 minutes, stained with 1% uranyl acetate in 50% alcohol for 15 minutes and washed in drops of distilled water three times for 5 minutes. The last drops of water were removed from the grids. The stained grids were air-dried. Images were obtained using an FEI/Philips TEM 208S microscope (Eindhoven, Netherlands) operating at an accelerating voltage of 100 KV. 

### Western blot analysis of plasma-derived exosomes

Exosomes were lysed in cell lysis buffer (RIPA buffer; CytoMatin Gene, Iran) supplemented with protease inhibitors (Sigma FAST™, USA) on ice. The concentration of exosomal lysates was determined using BCA Protein Assay Kit (Parsons Biotechnology, Iran). Approximately 50 µg of exosomal lysates were loaded per well. Proteins were separated on a 12% gel (Bio-Rad, UK) and transferred to nitrocellulose membranes (Bio-Rad, UK). To prevent non-specific binding, the membranes were blocked with 2.5% bovine serum albumin (BSA, CytoMatin Gene, Iran) powder diluted in tris-buffered saline-Tween (TBS-T), for 2 hours at room temperature (RT). 

The membranes were then incubated with monoclonal anti-CD63 (rabbit IgG, diluted 1:1000; System Biosciences, USA), mouse monoclonal CD81 (TAPA-1; clone 5A6, diluted 1:1000; Bio-Legend, USA) and rabbit polyclonal anti- Histone H3 (clone poly6019, diluted 1:500; Bio-Legend), as negative control overnight at 4°C. All antibody dilutions were made in TBS-T supplemented with 0.5% BSA. After incubation with primary antibodies, membranes were washed for 3×10 minutes in TBS-T (used for each wash step). The membranes were incubated with secondary horseradish peroxidase (HRP)-conjugated antibodies for 2 hours at RT. The secondary antibodies were goat anti-rabbit HRP IgG (diluted 1:20,000; System Biosciences, USA) and goat anti-mouse IgG (H+L, diluted 1:3000; Bio-Rad, UK). The membranes were washed 3×10 minutes. Finally, the signals were visualized using the ECL Western blotting kit (CMG, Iran), according to the manufacturer’s instructions. 

### Protein quantification of plasma-derived exosome or exosomal lysates

Five to ten microliters of plasma-derived exosome or exosomal lysate was dispensed into the wells of a 96-well plate. Then, the assay was performed by BCA Protein Assay Kit (Parstous Biotechnology, Iran) according to the manufacturer’s protocol. Protein content of the exosome lysates and exosomal total protein concentration were determined using a linear standard curve. A series of BSA was used to develop a standard curve.

### Isolation, purification, and expansion of natural killer cells

Blood samples were collected from refractory/relapsed patients, responsive patients and healthy donors. PBMCs were separated on a Ficoll-Hypaque gradient. NK cells were purified by negative selection, using NK cell isolation kit and LS columns (MiltenyiBiotec, Germany). The purity of N cells was confirmed as 85-90% by flow-cytometery (BD Company, USA) using PE-cy5labeled anti-CD56 and FITCI-labeled anti-CD3 (both from eBioscience, USA). The range of CD3 positive cell contamination in purified NK cells was 10-15%. 

To obtain polyclonal NK cell populations, PBMCs were.-ray irradiated (25 Gy) and they were used as autologousfeeder cells for co-culture with NK cells at a ratio of 4:1 feeder-NK cell. NK cells were expanded in Cellgro SCGMserum-free media (CellGenix, USA) supplemented with 5% human AB serum, 10% fetal bovine serum (FBS, Gibco, USA), 50 U/ml penicillin, 50 µg/ml streptomycin, 500 IU/ ml recombinant human interleukin-2 (IL-2, MiltenyiBiotecAG, Germany), 10 ng/ml recombinant human interleukin-15(IL-15, MiltenyiBiotec AG, Germany) at a density of 5×10^5^ cells/ml in T-25 flask for 3 weeks.

### Treatment of natural killer cells with plasma-derived exosomes

NK cells were seeded in 24-well plates at a density of 4×10^5^ cells per well in DMEM/F12 culture medium without FBS/AB serum. NK cell from healthy donors and DLBCL patients were treated with 20 µg plasma-derived exosomes of DLBCL patients (refractory/relapsed or responsive patients) at 37°C for 20 hours. Control wells contained no exosomes.

### RNA isolation and cDNA synthesis

Total RNA was extracted from NK cells using the miRCURY™ Isolation Kit-Cells (Exiqon, Denmark). Then, total RNA was quantified and converted to cDNA using the Universal cDNA Synthesis Kit II (Exiqon, Denmark) according to the following protocol: firstly, total RNA was incubated for 60 minutes at 42°C. Next, the reaction was followed by heat-inactivation of the reverse transcriptase for 5 minutes at 95°C. In addition, synthesis of cDNA was done by Thermo Scientific RevertAid First Strand cDNA Synthesis Kit (Fermentas, Thermo Fisher Scientific Inc., USA) according to the following protocol: first, total RNA and oligo (dT) 18 were incubated for 5 minutes at 65°C and they were next chilled on ice. Then the mixture of 5x Reaction Buffer, RiboLock RNase inhibitor, 10 mM dNTP mix and ReverAID M-MuLVRT was performed and it was incubated for 5 minutes at 25°C, followed by incubation for 60 minutes at 42°C. Ultimately, the reaction was terminated by incubation at 70°C for 5 minutes. 

### Quantitative reverse transcription-polymerase chain reaction for *hsa-miR-155-5p* and *hsa-let-7g-5p* as well as *SOCS-1* and *INPP5D*: gene expression assay

We used pre-designed primers (Exiqon, Denmark) for *hsa-let-7g-5p, hsa-miR-155-5p* and *SNORD44* (as reference gene). For mRNA quantification, specific primers were designed for *SOCS-1* and *INPP5D* (Pishgaman, Iran), using Allele ID software and BLAST (NCBI online server). Details of the primers are as following: 

GAPDH

F: 5´-CCA GTG GAC TCC ACG ACG TA-3´

R: 5´-ACT AAA ACC TCC CTA GAG CG-3´

SOCS-1

F: 5´-GTA GGA GGT GCG AGT TCA GG-3´

R: 5´-GAC CCC TTC TCA CCT CCT GA-3´

INPP5D

F: 5´-AAG CCT GTT GTC GTC CAT TG-3´

R: 5´-AGA CTC TGC CTT CAC CTC AAA-3´

All quantitative reverse transcription -polymerase chain reaction (qRT-PCR) reactions were performed duplicately at a final volume of 10 µl per well, using a 2x Real-Time PCR Master Mix (BioFACT™, Korea) and StepOne Plus™ quantitative real-time PCR detection system (Applied Biosystems, Thermo Fisher Scientific Inc.). The following thermal cycling conditions were applied: polymerase activation/denaturation at 95°Cfor 15 minutes, 45 amplification cycles at 95°C for 20 second, 60°C for 20 seconds and 72°C for 30 seconds. Threshold values for the threshold cycle determination (Ct) were generated automatically by the Step One Software v2.3 software. The microRNA and mRNA fold changes were determined compared to the control samples. Relative quantification method was employed, where the ΔΔCt value was obtained by analyzing difference between ΔCt of the sample and ΔCt of the calibrator (no exosomes).

### Measurement of interferon-gamma by ELISA

The culture supernatant was collected from NK cell medium treated with or without plasma-derived exosomes after 72 hours. The culture supernatant was stored in a -80°C freezer until assessment of the cytokine. Concentrations of IFN-γ in the culture supernatant were measured using the Human IFN-γ ELISA MAX™ Deluxe (Bio-Legend, USA). All procedures were performed according to the manufacturer’s instructions. 

### DLBCL patients’ plasma-derived exosomes effects on the rate of natural killer cells expressing CD16, NKG2D and CD69

NK cells were seeded into 96-well plates at a density of 1×10^5^ cells per well in DMEM/F12 culture medium without FBS/AB serum. Then, NK cells were treated with or without 20 µg (21) plasma-derived exosomes of refractory/relapsed or responsive patients with DLBCL at 37°C, 5% CO2 for 24 hours. NK cells were harvested and the percentage of NK cells expressing CD16, NKG2D and CD69 was determined by flow-cytometry, followed by comparing them in DLBCL patients and healthy donors. 

The following anti-human monoclonal antibodies were used for flow-cytometry: CD16 monoclonal antibody (B73.1, PE); CD314 (NKG2D) monoclonal antibody (1D11, PE); CD56 (NCAM) monoclonal antibody (CMSSB, PE); CD3 monoclonal antibody (OKT3, FITC) and CD69 monoclonal antibody (FN50, FITC; all purchased from eBioscience™). The cells were also stained with their corresponding isotype-matched control mAbs (Bio-Legend, USA). All samples were analyzed using the BD FACS Calibur system (Becton Dickinson Co., USA). Flowing Software version 2.5.1 (TerhoPerttu, Finland) was used for data acquisition and analysis. 

### Proliferation assay

Carboxyfluorescein succinimidyl ester (CFSE) was prepared as 5 mg/ml stocks in dimethyl sulfoxide (DMSO) and stored at -20°C. NK cells isolated from DLBCL patients and healthy donors were washed with PBS and resuspended in PBS (1×10^6^ cells/ml). NK cells were labeled with CFSE according to the protocol of CFSE Cell Division Tracker Kit (final concentration of 5 µM, Bio-Legend, USA) and incubated for 20 minutes at RT. After stopping the reaction with BSA in PBS (0.1% w/v), NK cells were washed and cultured in the absence or presence plasma-derived exosome of DLBCL patients (refractory/relapsed or responsive patients). Proliferation was analyzed after three days. 

### Natural killer cytotoxicity assay 

NK cells obtained from healthy donors and DLBCL patients, were co-incubated for 24 hours in the presence or absence of 20 µg exosomes isolating from patients with DLBCL. Treated NK cells (effector cells) were co- cultured with 50,000 CFSE labeled K562 target cells at different effector-to-target (E:T) ratios from 8:1, 4:1, 2:1 and 1:1 in 96-well plates containing 150 µl culture media. Target cells, including K562 cell lines, were labeled with CFSE according to the protocol of CFSE Cell Division Tracker Kit (final concentration of 5 µM, Bio-Legend) to discriminate target cells from effector cells. K562 and NK cells were co-cultured in DMEM/F12 mediumsupplemented with 500 U/ml IL-2 and 150 ng/ml IL-15(MiltenyiBiotec AG, Germany) for 4 hours at 37°C. Thesecells were stained with 0.05 µg of 7-amino-actinomycinD (7-AAD, Bio-Legend, USA) for 10 minutes in dark. Furthermore, the following control samples were prepared: unstained effector cells, unstained target cells, CFSE-stainedtarget cells, target cells stained with both CFSE and 7-AAD, target cells permeabilized with 5% (v/v) Triton X-100 TM(Sigma, USA) in PBS and stained with 7-AAD. Flowcytometry data were acquired from a FACS Calibur flowcytometer (Becton Dickinson Co.) and they were analyzed byFlowing Software version 2.5.1 (TerhoPerttu, Finland). NKcytotoxicity rate was calculated as the percentage of specificlysis using the following formula: (% of target cell lysis in thetest-% of spontaneous cell death)/(% of maximum lysis-% ofspontaneous cell death). Spontaneous cell death was obtained from target cells in the medium cultured alone. To obtain maximum cell lysis rate, target cells were treated with 5% Triton-X100. 

### Statistical analysis

Data were summarized by descriptive statistics: mean ± standard error (SE). Statistical analyses were performed using one-way analysis of variance (ANOVA) test for comparison among three groups (healthy donors, responsive or refractory/relapsed patients with DLBCL). In addition, one-way ANOVA test was used to determine statistically significant differences between NK cells treated with exosomes and untreated NK cells. Multiple comparisons of data were conducted using LSD post- hoc test between different treated groups. P<0.05 was considered statistically significant. IBM SPSS Statistics for Windows, version 21 (IBM Corp., Armonk, NY, USA) was used for data analysis. 

### Ethical considerations 

This study has been approved by the Bioethical Committee of the Isfahan University of Medical Sciences, Isfahan, Iran (IR.MUI.REC.1394.3.655/2015). The performed experiments comply with the current laws of Iran. All blood samples were taken from the patients after written informed consent and ethical permission was obtained for participation in the study. 

## Results

### Assessment of the plasma-derived exosome quality

The exosomes were identified based on their size, morphology and absence or presence of their specific proteins. Plasma-derived exosomes of the patients with DLBCL were evaluated by TEM (Fig .1B), Zetasizer (Fig .1C) and western blots (Fig .1D). TEM images showed spherical vesicles with morphological properties of the exosome and diameter of 50-150 nm. Moreover, Zetaseizer analysis demonstrated that the exosomes were spherical particles with a z-average diameter size of 90.18 nm. 

Exosomes identity was confirmed by Western blot analysis. When the lysed exosomes were probed with antiCD63 (tetraspanin, 54 KD) and anti-CD81 (tetraspanin, 26 KD), strong bands were detected on the blots. The morphology, size of <150 nm and presence of two CD63 and CD81 proteins, in the absence of Histone H3, strongly suggest that the studied vesicles were exosomes.

### Effect of plasma-derived exosomes on the expression level of microRNAs (*hsa-miR-155-5p* and *hsa-let-7g-5p*) and mRNAs (*SOCS-1* and *INPP5D*) 

Since *hsa-miR-155-5p* is critical for the homeostasis of NK cells, we investigated whether the expression level of this miRNA is up-regulated in the NK cells treated with plasma-derived exosomes obtaining from patients with DLBCL in comparison with untreated NK cells (14, 22). Therefore, we established a model *in vitro* system comprised of isolated exosomes co-incubated with human NK cells for 20 hours. Following co-incubation with exosomes, total RNA was extracted from NK cells, reverse transcribed and analyzed by qRT-PCR, as described in the "Materials and Methods" section. Changes in the expression levels of selected *hsa-miR-1555p, hsa-let-7g-5p* as well as, and *SOCS-1* and *INPP5D* were simultaneously measured in NK cells, relative to the control groups (no exosomes). 

**Fig.2:**
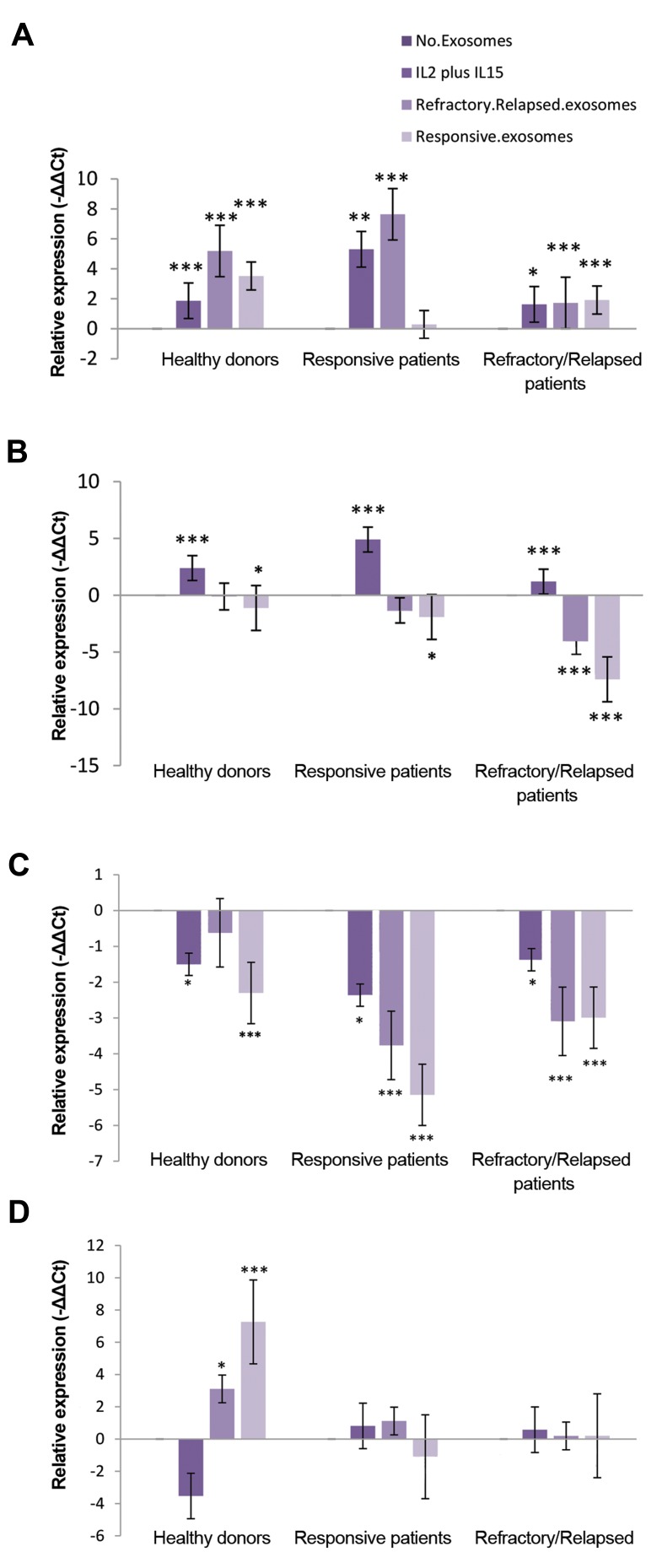
Effect of plasma-derived exosomes on the expression levels of microRNAs and mRNAs. The expression levels of A. hsa-miR-155-5p, B. hsa-let-7g-5p, C. SOCS-1 and D. INPP5D in the NK cells treated with IL-2/IL- 15 and plasma-derived exosome of patients with DLBCL compared to the untreated NK cells were determined in healthy donors, responsive DLBCL patients and refractory/relapsed DLBCL patients. NK cells were treated with 20 µg plasma-derived exosome of patients with DLBCL for 20 hours and they were then collected for preparation of total RNA. miRNAs (hsa- miR-155-5p and hsa-let-7g-5p) and RNA expression (SOCS-1 and INPP5D) were quantified by qRT-PCR. Degree of significance in treated NK cells with exosomes compared to the untreated NK cells was indicated by *P= 0.05, **P= 0.01 and ***P= 0.001 in each group. Each column shows mean of -..Ct ± standard error (SE). NK cells; Natural killer cells, IL; Interleukin, DLBCL; Diffuse large B-cell lymphoma, and qRT-PCR; Quantitative reverse transcription polymerase chain reaction.

Our results showed a significant increase in the expression levels of *hsa-miR-155-5p* of NK cells treated with plasma- derived exosomes of refractory/relapsed DLBCL patients compared to untreated NK cells in healthy donors, refractory/ relapsed DLBCL patients and responsive DLBCL patients (P=0.0001, LSD post-hoc test). Furthermore, a significant increase was observed in expression level of *hsa-miR-155-5p* in NK cells treated with plasma-derived exosomes of responsive DLBCL patients compared to untreated NK cells in refractory/relapsed DLBCL patients (P=0.009, LSD post-hoc test) and healthy donors (P=0.0001, LSD post-hoc test). The expression level of *hsa-miR-155-5p* in refractory/relapsed patients was lower than healthy donors and responsive patients in the presence of plasma-derived exosome of DLBCL patients (P=0.0001, Fig .2A). 

We observed a significant decrease in *hsa-let-7g-5p* expression level of NK cells treated with plasma-derived exosomes of refractory/relapsed DLBCL patients compared to untreated NK cells in refractory/relapsed DLBCL patients (P=0.0001, LSD post-hoc test). A significant decrease was observed in the expression level of *hsa-let-7g-5p* in NK cells treated with plasma-derived exosomes of responsive DLBCL patients compared to untreated NK cells in healthydonors (P=0.040), responsive DLBCL patients (P=0.042) and refractory/relapsed DLBCL patients (P=0.0001). In addition, there was a significant increase in the *hsa-let-7g5p* expression level of NK cells treated with IL-2/IL-15, compared to untreated NK cells in each group (P=0.0001, Fig .2B). The expression level of *hsa-let-7g-5p* for refractory/ relapsed patients was lower than healthy donors and responsive patients, in the presence of plasma-derived exosome of DLBCL patients (P=0.0001). 

Some studies showed the *SOCS-1* and *INPP5D* are two direct targets of *hsa-miR-155-5p* in many cell types. To determine whether plasma-derived exosomes of responsive or refractory/relapsed DLBCL patients are able to alter expression levels of *SOCS-1* and *INPP5D*, we examined these expression levels in NK cells of healthy donors and DLBCL patients. 

Our finding indicated that there was significant decrease in the expression levels of *SOCS-1* in NK cells treated with IL-2/IL-15 and plasma-derived exosomes of responsive DLBCL patients compared to untreated NK cells in healthy donors (P=0.016 and P=0.0001, respectively), responsive DLBCL patients (P=0.015 and P=0.0001, respectively) and refractory/relapsed DLBCL patients (P=0.014 and P=0.0001, respectively). Additionally, a significant decrease in the expression level of *SOCS-1* was observed in NK cells treated with plasma- derived exosomes of refractory/relapsed DLBCL patients compared to untreated NK cells in refractory/relapsed DLBCL patients (P=0.0001) and responsive DLBCL patients (P=0.0001, Fig .2C). 

There was a significant increase in *INPP5D* expression level of NK cells treated with plasma-derived exosomes of responsive or refractory/relapsed DLBCL patients compared to untreated NK cells in healthy donors (P=0.039 and P=0.0001, respectively). There was no significant difference in the *INPP5D* expression level of NK cells treated with plasma-derived exosome of DLBCL patients compared to untreated NK cells in refractory/ relapsed DLBCL patients and responsive DLBCL patients (Fig .2D).

### IFN-γ level in the natural killer cells culture supernatants in the presence of plasma-derived exosomeof DLBCL patients 

It was reported that *SOCS-1* and *INPP5D* negatively regulate IFN-γ production in NK cells. The IFN-γ concentration in culture supernatants of NK cells was determined by ELISA in the absence or presence of IL-2/IL-15 and plasma-derived exosomes of refractory/ relapsed patients and responsive patients after 72 hours of culture in an FBS-free or AB serum-free media. 

**Fig.3:**
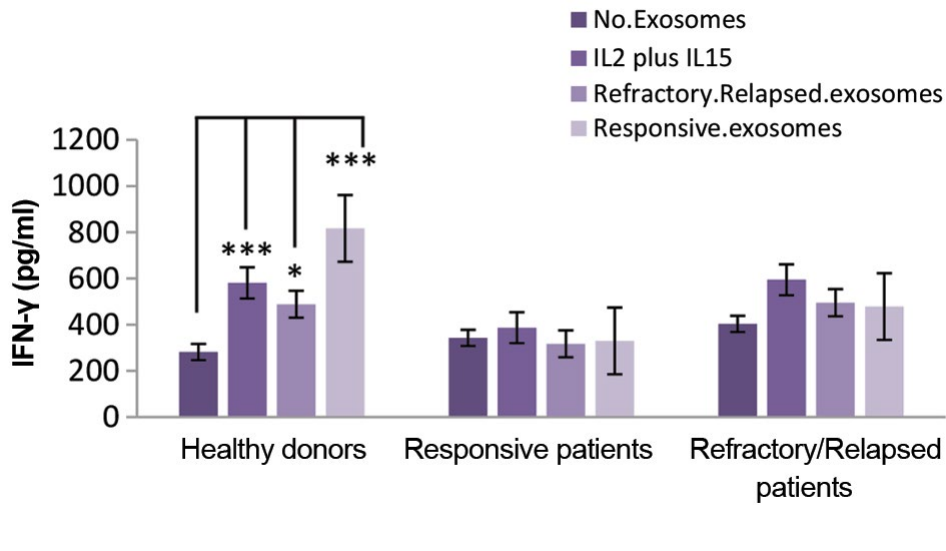
Effect of plasma-derived exosomes of responsive or refractory/ relapsed patients on the levels of IFN-γ. The media were harvested and IFN-γ concentration was measured by sandwich ELISA after 72 hours. Statistical relationships were determined in NK cells treated with IL-2/ IL-15, and plasma-derived exosomes of responsive or refractory/relapsed patients compared to untreated NK cells. Results were expressed as mean ± SE. *P<0.05, ***P<0.001 showed significant differences. IFN-γ; Interferon gamma, NK; Natural killer cells, and IL; Interleukin.

Our data showed increased level of *hsa-miR-155-5p* and decreased level of *SOCS-1* in healthy donors and DLBCL patients. A significant increase was observed in the cultured supernatant IFN-γ concentration of NK cells treated with IL-2/IL-15, plasma-derived exosome of responsive patients and plasma-derived exosome of refractory/relapsed patients compared to untreated NK cells in healthy donors (P=0.0001, P=0.01 and P=0.0001, respectively). However, there was no significant difference in the culture supernatant IFN-γ concentration of NK cells treated with IL-2/IL-15 and plasma-derived exosome of *DLBCL* patients compared to the untreated NK cells in responsive patients or refractory/relapsed patients. The data are shown in Figure 3. 

In addition, IFN-γ concentration in the culture supernatant of NK cells from refractory/relapsed DLBCL patients was lower than the responsive DLBCL patients in the presence of IL-2/IL-15, plasma-derived exosome of responsive patients and refractory/relapsed patients (P=0.002, P=0.001 and P=0.002, respectively). 

### Effects of plasma-derived exosome of DLBCL patients on the percentage of natural killer cells expressing CD16, CD69 and NKG2D 

**Fig.4:**
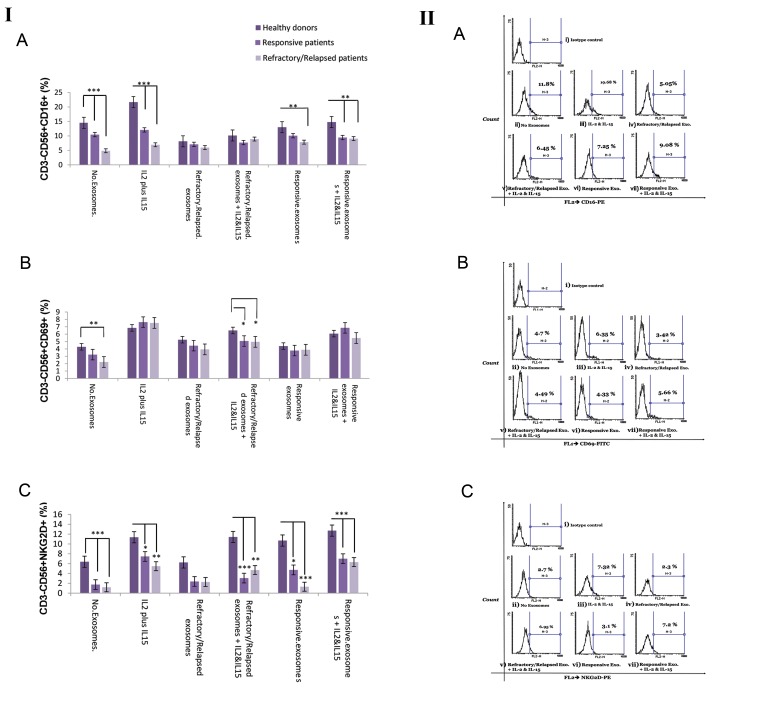
Flow cytometer analysis of NK cell surface markers (CD16, NKG2D, and CD69) in the absence or presence of plasma-derived exosome of DLBCL patients. I. These surface markers were analyzed by gating on the live NK cells (CD56+CD3) of a representative DLBCL patient. A. NK cell labeled with PE-anti-human CD16 and PE Mouse IgG1, k Isotype control, B. NK cell labeled with FITC-anti-human CD69 and FITCI Mouse IgG1, k Isotype control, C. NK cell labeled with PE-anti-human CD314 (NKG2D) and PE Mouse IgG1, k Isotype control, i. Isotype control, ii. Unstimulated NK cell, iii. IL-2/ IL-15, iv. Plasma-derived exosomes of DLBCL refractory/ relapsed patients, v. Plasma-derived exosomes of DLBCL refractory/ relapsed patients plus IL-2/IL-15, vi. Plasma-derived exosome of responsive DLBCL patients and vii. Plasma-derived exosome of responsive DLBCL patients plus IL-2/IL-15. II. Average of the percentage of NK cells expressing A. CD16, B. CD69 and C. NKG2D was determined in each group (responsive DLBCL patients and refractory/relapsed DLBCL). Degree of significance was indicated by *P<0.05, **P<0.01, ***P<0.001. Each bar illustrates the mean ± SE. NK; Natural killer cells and DLBCL; Diffuse large B-cell lymphoma

The percentage of NK cells expressing CD16, CD69 and NKG2D was determined by flow-cytometer in the absence or presence of IL-2/IL-15 and 20 µg of plasma-derived exosome of DLBCL patients in each group (healthy donors and responsive or refractory/relapsed patients with DLBCL) after 24 hours of culture in a FBS-free or AB serum-free media (Fig .4I).

Our findings showed that the percentage of CD16^+^ NK cells from healthy donors was more than refractory/relapsed DLBCL patients in the absence of exosomes or in the presence of IL-2/ IL-15, plasma-derived exosome of responsive DLBCL patients and plasma-derived exosome of responsive DLBCL patients plus IL-2/IL-15 (P=0.0001, P=0.0001, P=0.008 and P=0.001, respectively). Moreover, the results showed that percentage of the CD16^+^ NK cells from responsive DLBCL patients was more than refractory/relapsed DLBCL patients in the absence of exosomes or presence of IL-2/IL-15 (P=0.0001 and P=0.002, respectively). 

In addition, a significant reduction was observed in the percentage of CD16^+^ NK cells in the presence of plasma-derived exosomes of refractory/relapsed DLBCL patients in responsive DLBCL patients (P=0.02) and healthy donors (P=0.0001). A significant increase was observed in the percentage of CD16^+^ NK cells in the presence of IL-2/IL-15 in healthy donors (P=0.0001). 

The percentage of CD69^+^ NK cells from healthy donors was more than refractory/relapsed DLBCL patients in the absence of exosomes (P=0.003). The percentage of CD69^+^ NK cells from healthy donors was also more than refractory/relapsed DLBCL patients and responsive DLBCL patients in the presence plasma- derived exosome of refractory/relapsed DLBCL patients plus IL-2/IL-15 (P=0.018 and P=0.034, respectively, ANOVA test). 

Furthermore, there was a significant increase in the percentage of CD69^+^ NK cells in the presence IL-2/IL15 compared to the absence of exosomes in refractory/ relapsed DLBCL (P=0.038), responsive DLBCL patients (P=0.0001) and healthy donors (P=0.001). We also observed significantly increased CD69^+^ NK cell percentage in the presence plasma-derived exosome of refractory/relapsed DLBCL patients plus IL-2/IL-15 in comparison with the absence of exosomes in responsive DLBCL patients and healthy donors (P=0.014 and P=0.005, respectively, LSD Post-Hoc). In addition, there was an increased CD69^+^ NK cell percentage in the presence plasma-derived exosome of responsive DLBCL patients plus IL-2/IL-15 compared to the absence of exosomes in responsive DLBCL patients and healthy donors (P=0.0001 and P=0.022, respectively, LSD Post-Hoc). 

The percentage of NKG2D^+^ NK cells from healthy donors was more than DLBCL patients in the absence of exosomes or in the presence of IL-2/IL-15, plasma- derived exosome of refractory/relapsed DLBCL patients, plasma-derived exosome of refractory/relapsed DLBCL patients plus IL-2/IL-15 and plasma-derived exosome of responsive DLBCL patients (P<0.05, P<0.01 and P<0.001, ANOVA test). Data is presented in Figure 4II. 

There was no significant difference in the percentage of NKG2D^+^ NK cells in the presence of plasma-derived exosome of DLBCL patients in each group. There was significant increase in percentage of NKG2D^+^ NK cells in the presence of IL-2/IL-15 in comparison with the absence of exosomes in refractory/relapsed DLBCL patients (P=0.05), responsive DLBCL patients (P=0.0001) and healthy donors (P=0.014). We also observed significant increase in the percentage of NKG2D^+^ NK cells in the presence of plasma-derived exosome of responsive DLBCL patients plus IL-2/IL-15 in comparison with the absence of exosomes in responsive DLBCL patients (P=0.02) and healthy donors (P=0.003). There was a significant increase in the percentage of NKG2D^+^ NK cells in the presence of plasma-derived exosome of refractory/ relapsed DLBCL patients plus IL-2/IL-15 compared to the absence of exosomes in refractory/relapsed DLBCL patients (P=0.011) and healthy donors (P=0.028). 

### Effect of plasma-derived exosomes of DLBCL patients on natural killer cell proliferation 

**Fig.5:**
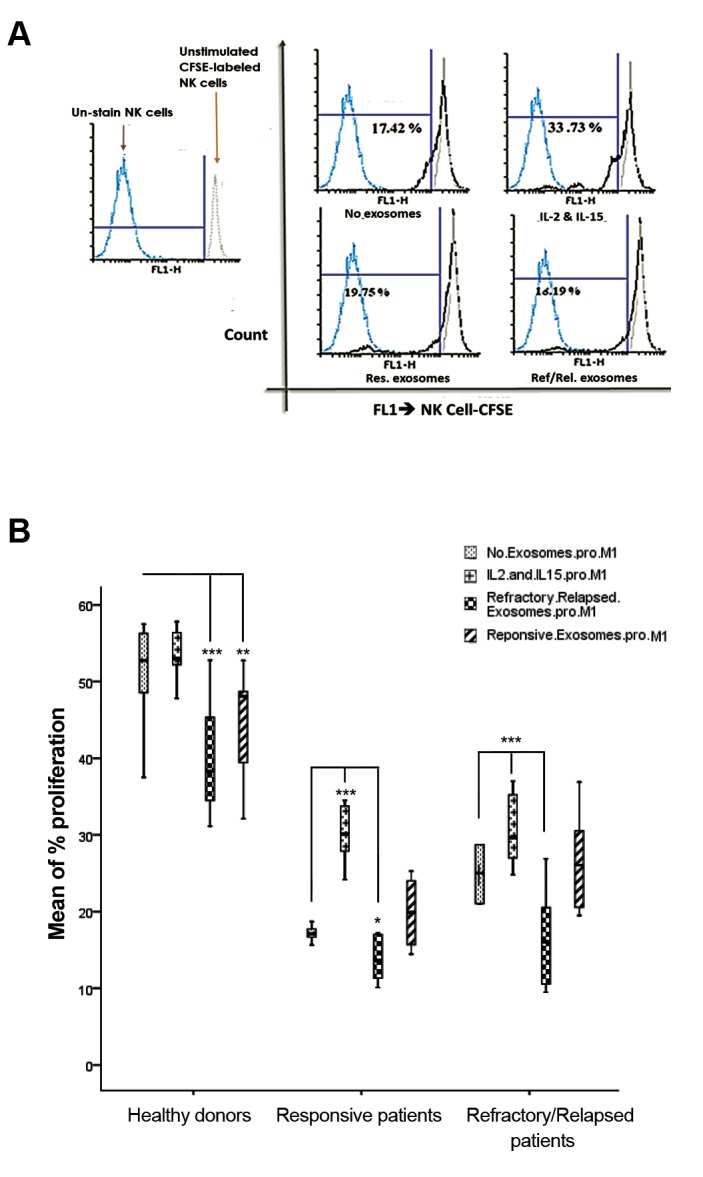
Effect of plasma-derived exosome of DLBCL patients on proliferation of labeled NK cell with CFSE. A. CFSE-positive NK cell population of a representative responsive DLBCL patient was cultured in the absence or presence of plasma-derived exosome from DLBCL patients, for three days. The dotted blue line represents unstained NK cells. Gray line with an empty profile in histograms indicates unstimulated CFSE-labeled NK cells and B. Degree of significance in the treated NK cells with plasma-derived exosomes of responsive or refractory/relapsed patients with DLBCL compared to the untreated NK cells, is indicated by *P=0.05, **P=0.01, and ***P=0.001 in each group. Each column illustrates the mean SE for proliferation rate of NK cells. DLBCL; Diffuse large B-cell lymphoma and NK; Natural killer cells.

We investigated whether plasma-derived exosomes of responsive or refractory/relapsed patients with DLBCL plays role in the proliferation of NK cells (Fig .5). Proliferation rate of NK cells from healthy donors was more than responsive DLBCL patients and refractory/ relapsed DLBCL patients in the absence of exosomes or in presence of IL-2/IL-15 as well as plasma-derived exosome of DLBCL patients (P<0.001). 

Furthermore, there is a significant increase in the proliferation of NK cells treated with IL-2/IL-15 in responsive patients (P=0.0001) and refractory/relapsed patients (P=0.007, Fig .5). Additionally, there was a significant decrease in the proliferation of NK cells treated with plasma- derived exosomes of refractory/relapsed patients compared to the untreated NK cells in healthy donors (P=0.0001), responsive patients (P=0.044) and refractory/relapsed patients (P=0.0001). A significant decrease was also determined in the proliferation of NK cells treated with plasma-derived exosome of responsive patients compared to untreated NK cells in healthy donors (P=0.009). 

### Effect of plasma-derived exosome of DLBCL patients on natural killer cell cytotoxicity 

**Fig.6:**
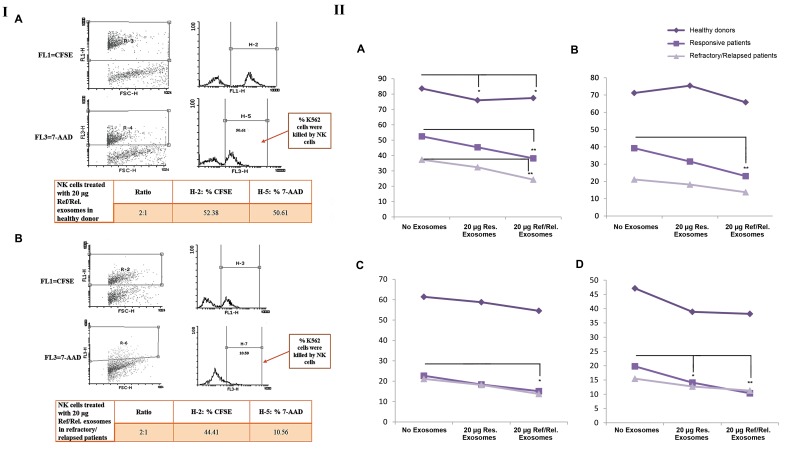
Effect of plasma-derived exosome of DLBCL patients on the NK cell cytotoxicity. I. CFSE-stained K562 cells were co-cultured with NK cells at different E/T ratios. The CFSE-stained K562 cells were first gated by FSC and SSC characteristics. Both dot plots and histograms show that CFSE-stained K562 target cells were killed by NK cells treated with 20 μg plasma-exosomes of refractory/relapsed patients in A. Healthy donor and B. Responsive patient using E/T ratio of 2:1. The numbers in the panels denote percentage of 7-AAD positive cells. II. Variety of these killing activities were statistically analyzed at different E/T ratios, including A. 8:1, B. 4:1, C. 2:1, and D. 1:1 in the absence or presence of plasma-derived exosome of patients with DLBCL in healthy donors, responsive DLBCL patients and refractory/relapsed DLBCL patients. Degree of significance is highlighted by *P≤0.05 and **P≤0.01 in each group. Each point illustrates mean of the NK cell cytotoxicity percentage in each group. DLBCL; Diffuse large B-cell lymphoma and NK; Natural killer cells.

NK cell-mediated cytotoxicity was measured after co- culture of K562 cells with untreated NK cells or NK cells treated with plasma-derived exosome of DLBCL patients at different effector-to-target (E:T) ratios (8:1, 4:1, 2:1 and 1:1). More than 92% of K562 cells were stained with CFSE and spontaneous lysis was in the range 1.23 -7.94 (Fig .6I). Our data showed that NK cells cytotoxicity (at ratios of 8:1, 4:1, 2:1 and 1:1) in the absence or presence plasma-derived exosome of DLBCL patients in healthy donors was more than DLBCL patients (P=0.0001, ANOVA test). 

There was a significant decrease in NK cell- mediated cytotoxicity treated with plasma-derived exosomes of refractory/relapsed DLBCL patients compared to untreated NK cell in refractory/relapsed DLBCL patients [at ratios of 8:1 (P=0.001), 4:1 (P=0.003), 2:1 (P=0.021) and 1:1 (P=0.001)] and in refractory/ relapsed DLBCL patients [at 8:1 ratios (P=0.001)]. In addition, a significant decrease was observed in NK Cell-mediated cytotoxicity treated with plasma-derived exosome of responsive patients with DLBCL compared to untreated NK cell in responsive patients (at 1:1 ratio, P=0.033). The results of cytotoxicity assay are presented in Figure 6II. As well, there was the significant decrease in NK cells-mediated cytotoxicity treated with plasma- derived exosomes of refractory/relapsed DLBCL patients (P=0.03) and plasma-derived exosomes of responsive DLBCL patients (P=0.01) in healthy donors at ratio of 8:1. 

## Discussion

Several reports have indicated that tumor-derived exosomes down-regulate signaling in NK cells (23). The exact nature of the signals delivered via exosomes and the mode of action are unknown (22). Studies reveal that regulation of NK cell activation by *hsa-miR-1555p* is complex and *hsa-miR-155-5p* can function as a dynamic tuner for NK cell activation (24). We considered the possibility that plasma-derived exosome of patients with DLBCL can cause some effects on the *miR-155* IFN-γ pathway in NK cells as well as proliferation and cytotoxicity of NK cells. The present study provides evidence of some signatures of plasma-derived exosome of patients with DLBCL on NK cell function. 

We showed a significant increase in proliferation, *hsa-let-7g-5p* level as well as the percentage of CD69^+^ and NKG2D^+^ NK cells in the presence of IL-2/IL-15. Findings obtained from the current investigation was consistent with previous studies reporting that IL-15 and IL-2 stimulate proliferation and activation of NK cells (25). In this study, we observed significant decrease in the percentage of CD69^+^ NK cells, CD16^+^ NK cells and NKG2D^+^ NK cells, IFN-γ production, NK cell proliferation and cytotoxicity in the absence or presence of IL-2/IL-15 in refractory/relapsed patients compared to responsive patients and healthy donors. Furthermore, we found that exposure of NK cells from healthy donors in the presence of 20 µg exosomes, isolated from DLBCL patients, increased *hsa-miR-155-5p* and IFN-γ levels and reduced NK cells proliferation. Moreover, the exposure of NK cells from patients with DLBCL in the presence of 20 µg exosomes, isolated from refractory/relapsed DLBCL patients, increased *hsa-miR-155-5p* level and reduced proliferation and cytotoxicity of NK cells.

Our finding showed a significant increase in *hsa-miR155- 5p* level and a significant decrease in SOCS-1 level in NK cells treated with 20 µg plasma-derived exosome of DLBCL patients in comparison with the untreated NK cell in healthy donors. Additionally, we observed an increased level of *hsa-miR-155-5p* in association with increased level of IFN-γ, in the presence of plasma- derived exosome of DLBCL patients in healthy donors. These results were consistent with the previous studies. These studies report that *hsa-miR-155-5p* is a positive regulator of IFN-γ production. The cytokine-induced up- regulation of *hsa-miR-155-5p* enhances IFN-γ production by targeting and suppressing *INPP5D* and *SOCS-1* (as the negative regulators), in the activated NK cell through cytokines (IL-12 and IL-18) and CD16 (8, 26). 

An increased level of *hsa-miR-155-5p* and a decreased level of *SOCS-1* were observed in the presence of 20 µg plasma-derived exosome of DLBCL patients, in patients with DLBCL. Nevertheless, no significant difference was observed in the *INPP5D* and IFN-γ expression levels in the presence of 20µg plasma-derived exosomes of responsive or refractory/relapsed patients, in DLBCL patients. Therefore, plasma-derived exosome of DLBCL patients may carry or target other microRNAs (*has-miR- 29, hsa-miR-155-5p* and *has-miR-15/16*) or other upstream pathways regulating IFN-γ level in NK cells of the DLBCL patients (27).

Although we observed significant increase in *INPP5D* level, a significant increase was determined in IFN-γ level produced by NK cells treated with plasma-derived exosome of DLBCL patients in healthy donors. These findings were contrary to the previous studies. Since mRNA may undergo post-transcriptional modifications, quantification in the both mRNA and protein levels are necessary to understand how the cells work in different condition (28). Therefore, we should evaluate *INPP5D* and *SOCS-1* expressions in the levels of mRNA and protein to find the effect of exosomes isolated from patients on IFN-γ production. 

We observed a decreased level of *hsa-let-7g-5p* in NK cells, treated with 20 µg plasma-derived exosome of responsive DLBCL patients in comparison with untreated NK cell in healthy donors and DLBCL patients. These results explain unknown factors, in the exosomes, which could contribute to the reduction of *hsa-let-7g-5p* level. A report showed that decreased level of *hsa-let-7g-5p* associated with a higher risk of tumor relapse in patients with advanced pathological stage of gastric and breast cancers (29). Some studies suggest that low expression levels of *hsa-let-7g-5p* have a longer event free survival time (30). In other word, some evidence demonstrates that *hsa-let-7g-5p* can suppress NF-κB signaling pathways and secretion of pro-inflammatory cytokines, while *hsa-miR155- 5p* up-regulates NF-κB through down-regulation of IKKs and other genes (31-33). Thus, decreased expression level of hsa-let-7g-5p and increased expression level of *hsa-miR-155-5p* in the presence of exosome isolated from patients might be associated with up-regulation of NF-κB in NK Cells. It is necessary to investigate roles of *hsa-miR- 155-5p* and *hsa-let-7g-5p* in NF-kB pathway, in the absence or presence of exosome isolated from patients, in PBMCs obtained from DLBCL patients. 

We investigated NK cells proliferation after three days and a significant decrease was observed in NK cells treated with plasma-derived exosome of refractory/ relapsed DLBCL patients compared to untreated NK cells in three groups. Some reports have shown that the exosomes isolated from tumor cell supernatants and patients’ sera inhibit proliferation of CD8^+^ T-cells (21, 34). Clayton et al. (35) indicates that tumor exosomes inhibit IL-2 mediated lymphocyte proliferation (50%) in purified CD4+ T-cell population. However, in the presence of tumor exosomes, NK cell proliferation has only been slightly decreased. They revealed that exosomesassociated transforming growth factor-ß1 (TGF-ß1) contributed to anti-proliferative effects. This reduction might be due to the presence of TGF-ß1 or other anti- proliferation agents in plasma-derived exosomes of refractory/relapsed DLBCL patients. On the other hand, we observed that plasma-derived exosomes of refractory/ relapsed DLBCL patients decreased expression levels of SOCS-1 and NK cell proliferation in DLBCL patients. The results of this study are in line with another study in the mouse model. They report that *miR-155-5p* containing exosomes produced by macrophage under stress, suppress proliferation of the fibroblast by down-regulation of SOCS-1 protein expression (36). Thus, a decrease in the expression levels of SOCS-1 might result in a decrease in NK cell proliferation. In addition, an increased level of *hsa-miR-155-5p* and decreased levels of *SOCS-1* and *hsa-let-7g-5p* in the presence of plasma-derived exosome of DLBCL patients might result in an increase of inflammation. However, we did not investigate effect of inflammatory cytokines, such as IL-6 and TNF-ß. 

A study showed that decreased CD16 expression level in the NK cells of patients with DLBCL can lead to the impairment in rituximab-mediated ADCC (37). We observed significant decreased percentage of CD16^+^ NK cells in the presence of 20 µg plasma-derived exosome of refractory/relapsed patients in responsive patients and healthy donors. This finding showed that plasma-derived exosome of refractory/relapsed patients might impair ADCC. 

No significant difference was found in the percentage of CD69^+^ or NKG2D^+^ NK cells in the presence of plasma- derived exosome of DLBCL patients in each group. Contrarily, some studies demonstrated that NKG2D expression is down-regulated by micro-vesicles or exosomes, associated with TGF-ß1 and IL-10 and/or exosomes bearing NKG2D ligands. Down-regulation of NKG2D surface protein causes decreased ability of NK cells to recognize malignant cells (10, 37, 38). In addition, a study has described that exosome of cancer patients mediated higher immune suppression by reducing CD69 expression in activated CD4+ T effector cells after 7 hours (39). These results might be due to the small sample size, using no FBS or AB serum for NK cell culture, incubation time and sample type (responsive DLBCL patients vs. refractory/relapsed patients). 

A recent review study explained that tumor-derived exosomes inhibit NK cell activation, cytotoxicity and proliferation. In fact, these exosomes bear TGF-ß1 or apoptosis-inducing ligands (Fas ligand and TNF-related apoptosis-inducing ligand). Therefore, they can initiate T cell apoptosis or disrupt IL-2 signaling in NK cells (40). Similarly, we observed a significant decreased NK cell cytotoxicity, in the presence of plasma-derived exosome of DLBCL patients, in DLBCL patients. However, the exposure of NK cells from healthy donors, in presence of 20 µg plasma-derived exosome of DLBCL patients, did not have any effects on NK cell cytotoxicity. Therefore, it was better to evaluate cytotoxicity of NK cells against DLBCL cell lines to understand the main effect of exosomes released from DLBCL cell line on NK cell function. Disruption in the cytotoxic machinery of NK cells might also result from down-regulation of NKG2D expression. However, we did not observe any significant difference in the percentage of NKG2D^+^ NK cells, in the absence or presence of plasma-derived exosome of DLBCL patients. 

## Conclusion

To sum up, the importance of NK-cell in removing hematopoietic cancer provides a strong rationale to use NK-cells therapy instead of autologous stem cell transplantation for treatment of refractory/ relapsed patients with DLBCL. Our report indicates decreased percentage of CD16^+^CD69^+^NKG2D^+^ NK cells, low IFN-γ levels in the supernatant of NK cell cultures, decreased NK cell proliferation and reduced NK cell cytotoxic activity in DLBCL patients compared to the healthy donors in the absence plasma-derived exosome of DLBCL patients. This could become the foundation of new therapeutic agent developments to target the NK cell activation and NK cell cytotoxicity. Our findings demonstrated decreased proliferation and cytotoxicity of NK cell in the absence or presence of plasma-derived exosome of DLBCL patients. It seems that elimination of plasma-derived exosome of patients using some drugs and also other procedures could be a great way to improve NK-cell functions. Ultimately, use of dendritic cell-derived exosomes and NK cell- derived exosomes might be helpful as cell-free cancer vaccines in the clinical setting.
